# Protein Detection of Excretory-Secretory Products and Somatic Extracts from *Fasciola hepatica* and *F. gigantica* Using Two-Dimensional Electrophoresis

**Published:** 2019

**Authors:** Afshin RASOULI, Ali FARAHNAK, Hakimeh ZALI, Mostafa REZAEIAN, Abolfazl GOLESTANI, Mohammad Bagher MOLAEI RAD

**Affiliations:** 1. Department of Parasitology and Mycology, School of Public Health, Tehran University of Medical Sciences, Tehran, Iran; 2. Proteomics Research Center, School of Advanced Technologies in Medicine, Shahid Beheshti University of Medical Sciences, Tehran, Iran; 3. Department of Biochemistry, School of Medicine, Tehran University of Medical Sciences, Tehran, Iran

**Keywords:** Fascioliasis, Excretory-secretory materials, Somatic products, Two-dimensional electrophoresis, *Fasciola*

## Abstract

**Background:**

The aim of this research was to compare excretory-secretory and somatic extract materials of *Fasciola hepatica* and *F. gigantica* to detect protein maps of two species.

**Methods:**

Twenty infected livers were collected from sheep in industrial slaughterhouse in Tehran, 2017–2019. Worms were detached from bile ducts, then recognized according to morphologic and morphometric criteria. After three times washing, worms were incubated in RPMI culture media and excretory-secretory products were collected. Worms were crushed and homogenized for preparation of somatic extract. Two Dimensional Electrophoresis gels were accomplished for both excretory-secretory material and somatic extracts. Gels were scanned with densitometer and analyzed with Same Spots software and protein spots were identified with Expasy database.

**Results:**

For both excretory-secretory products and somatic extract, protein spots were appeared with two-dimensional electrophoresis technique. Quantitative analysis showed 40 and 28 protein spots for excretory-secretory of *F. hepatica* and *F. gigantica* respectively. For somatic extract 19 and 12 protein spots were recognized for *F. hepatica* and *F. gigantica* in that order.

**Conclusion:**

The rate of expression of some proteins were more in *F. hepatica* while expression of other proteins was high in *F. gigantica*. The expression of protease enzyme was higher in *F. gigantica* than *F. hepatica*. These data could be considered for biochemical differentiation of *Fasciola* species and subsequently to design and prepare of antigens for diagnosis/vaccine development.

## Introduction

Among helminthic diseases, fascioliasis is a common illness and from the economic lost and public health disorder point of view is very important. Both two species in *Fasciola* parasites (*Fasciola hepatica* and *F. gigantica*) are responsible for acute and chronic form of disease (1). In these parasites, attacking, settlement, nutrition, migration, reproduction and pathogenesis depend to several biomolecules which between them, proteins are one of the most vital elements (2). Isolation and detecting of proteins help to perception of biological and biochemical interaction between host and parasites and reactions in the parasite itself (3).

There are several methods for detecting and studying proteins which among them Double Dimension Electrophoresis is common and practical technique. Isoelectric Focusing Electrophoresis (IEF) separates proteins based on their isoelectric point. In this method, proteins move to one pole and at the isoelectric point accumulate in the special point of the gel (4). Subsequently, electrophoresis would be continued using SDS-PAGE as second dimension. In this step, the proteins separate based on their molecular weight and after staining different proteins appear as spots in the gel (5). SDS-PAGE technique has been used to compare ES and Somatic extract in *F. hepatica* and *F. gigantica* (6). Double Dimension Electrophoresis (2DE) and Mass Spectrometry were used for comparison of proteome between ES products of *F. hepatica* in vitro and in vivo (7).

The aim of research was to compare proteins in ES and somatic extract materials of *F. hepatica* and *F. gigantica* to detect protein maps of two species.

## Materials and Methods

### The collection of parasites

Twenty infected livers of sheep were collected from an industrial slaughterhouse (Saman, Tehran, Iran, 2017–2019) and transferred to Tehran University of Medical Sciences, Helminthology Laboratory. *Fasciola* species were detached from bile ducts and differentiated according to morphologic and morphometric criteria. *F. gigantica* has greater length, larger ventral sucker, shorter cephalic cone and wider end of body than *F. hepatica* (8).

### The preparation of excretory-secretory products of parasites

After 3 times washing with phosphate buffer saline (PBS, pH 7.4), worms were incubated in small chambers contain at least 1 ml RPMI 1640 Medium under 5% CO_2_ for 6 h. Culture solutions were centrifuged at 4 °C in 10000 gr for 15 min and supernatants were stored in − 20 °C until required (9).

### The preparation of somatic extractcs of parasites

To prepare somatic extract, worms were crushed and homogenized in PBS buffer with a sterile pestle and mortar at 4 °C on ice. After centrifuging in 4 °C and 10000 gr for 30 min, the supernatant of solutions were collected and preserved until required (10).

### Two-dimensional electrophoresis of samples

Two-dimensional electrophoresis was accomplished for both ES materials and somatic extracted solutions of *Fasciola* species. For first dimension, IEF tubes were washed with sulphochromic acid, and then rinsed with distilled water. Tubes were immersed in alcoholic KOH, rinsed with distilled water three times, and dried. IEF tubes were filled with gel solutions and left till polymerized. Gel gradient was done by 30 min at 200, 15 min in 300 and 15 min in 400 volt of currency. Finally, specimens were run into gels and protein bands were gained in the IEF gels (11, 12). For second dimension, SDS-PAGE Electrophoresis, glasses of electrophoresis were washed with detergent to remove fat and other pollutants, rinsed with distilled water three times, dried and were cleaned with ethanol. Casts were tightened to tank body and sealing gel was poured into chamber. After sealing, resolving gel were poured and 30 to 45 min were given until polymerized. Stacking gel was adjusted above previous gel. After polymerizing, IEF gels, which 30 min were floated in equilibration buffer, were laid on SDS gel beside a proper protein marker. Power supply was adjusted in 100 volts, after approximate 6 hours; the gels were transformed into coomassie blue stain for at least 15 min. After appearing of protein spots, the gels were destained in fixative (11–13).

### Protein identification

Prepared gels from ES and somatic products were scanned with densitometer and analyzed with SameSpots software and protein spots were identified with Expasy Protein Database (14).

## Results

**Fig. 1:**
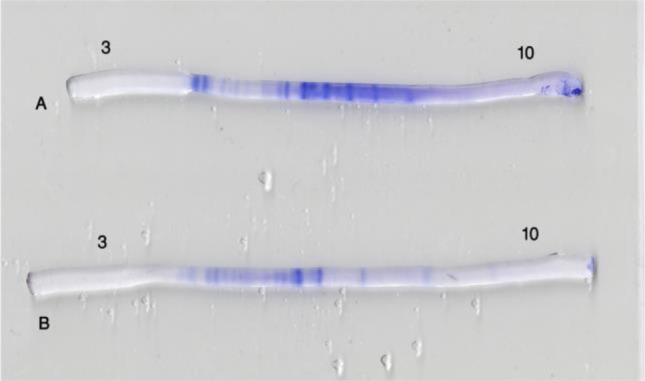
Determined protein bands in the ES materials of *F. hepatica* (A) and *F. gigantica* (B) in IEF gel. Protein bands between pH 6.5–7.5 in A are more than B

**Fig. 2:**
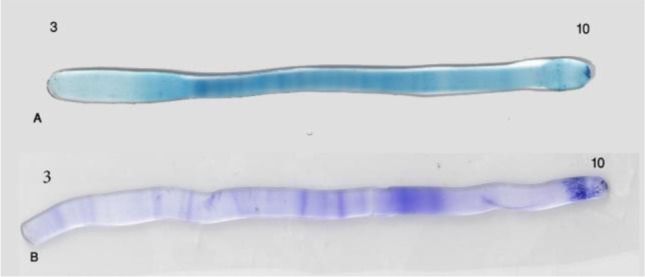
Determined protein bands in the somatic materials of *F. hepatica* (A) and *F. gigantica* (B) in IEF gel. Protein bands between pH 4.5–5 in A are more than B

Protein bands of ES and somatic extract of *F. hepatica* and *F. gigantica* were appeared on IEF gels (Figs. 1–2).

**Fig. 3:**
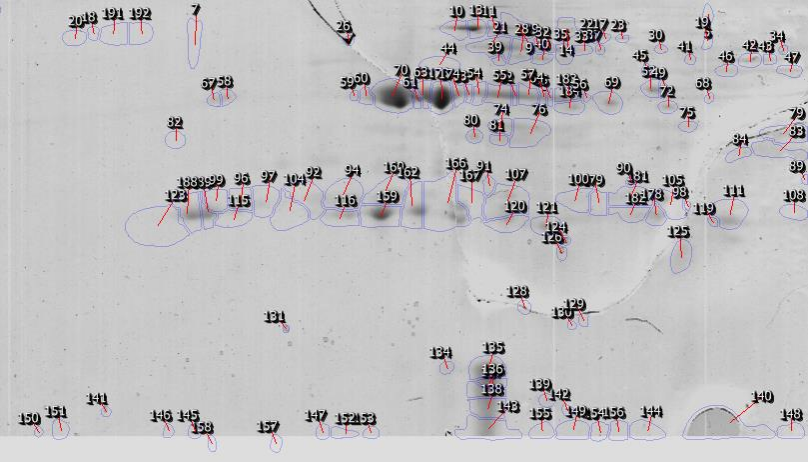
Comparison of protein spots in the reference image of ES materials of *F. hepatica* and *F. gigantica* in 2-DE gel. Expression of NADH ubiquinone oxidoreductase spot (90), thioredoxin peroxidase spots (23 and 121), mitochondrial acetate succinase spot (75), calmoduline like protein 2 spot (131), leucyl aminopeptidase spot (173), cytrate synthase spot (184), galactokinase like protein spot (59), cytochrom isomers spots (70 and 81) and thioredoxin glutathione reductase spot (63) were identified in excretory-secretory of *F. hepatica* more than *F. gigantica*, however cathepsin L protein isomers spots (92, 104 and 166), ubiquinone oxidoreductase spot (182), vitelin protein B1 spot (105), Nucleotide Sensitive Chloride current inducer spot (188), triose Phosphate Isomerase spot (179) and calcium transporting ATPase spot (13) in *F. gigantica* were more than *F. hepatica* (*P*<0.05)

**Fig. 4:**
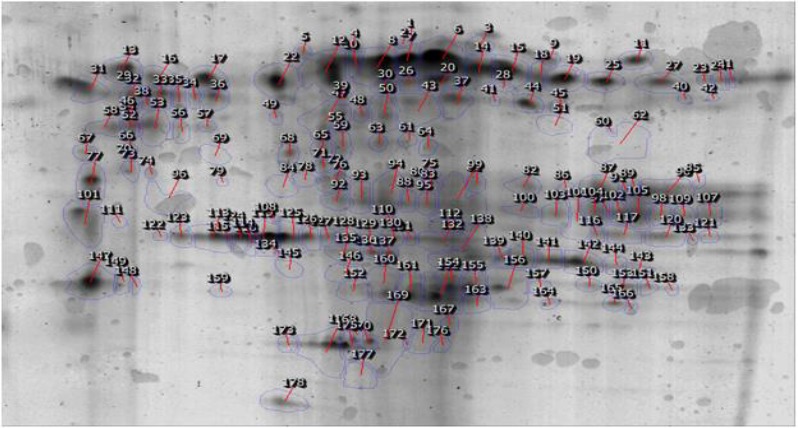
Comparison of protein spots in the reference image of somatic materials of *F. hepatica* and *F. gigantica* in 2-DE gel. Expression of calcium transporting ATPase isomer and thioredoxin glutathione peroxidase isomers spots (129 and 169) in *F. hepatica* were detected more than *F. gigantica* and expression of calcium transporting ATPase isomers spots (61 and 64) and thioredoxin glutathione reductase isomers spot (172) in *F. gigantica* were more than *F. hepatica* (*P*<0.05)

The comparison of protein spots in double dimension gel for ES materials and somatic extract of two species were showed in Figs. 3, 4.

Gels were scanned with densitometer and analyzed with SameSpots software and protein spots were determined. Proteins were identified using Expasy bioinformatics database and presented in tables 1–4. Quantitative analysis showed 40 and 28 protein spots for excretory-secretory of *F. hepatica* and *F. gigantica* respectively. For somatic extract 19 and 12 protein spots were recognized for *F. hepatica* and *F. gigantica* in that order. According to tables' data, the rate of expression of some proteins was more in *F. hepatica* while expression of other proteins was high in *F. gigantica*.

**Table 1:** Recognized proteins of the ES products of *Fasciola* spp. utilizing Expasy bioinformatics database (Expression of proteins in *F. hepatica* more than *F. gigantica* with 95% confidence interval/at the 5% significant level)

***pH***	***Molecular weight (kDa)***	***Fold***	***Spot number***	***Name of protein***
9.39	92	2.5	147	NR
6.27	171	2	20	NR
6.31	180	2.8	6	NR
8.52	110	1.7	123	NR
4.6	127	1.8	91	NR
9.45	169	2	31	NR
4.39	128	1.8	90	NR
6.55	179	2.1	7	NR
6.75	169	1.3	30	NR
8.11	111	3.7	115	NR
4.84	128	1.6	87	NR
7.05	178	2.1	10	NR
6.88	109	1.9	129	Calcium transporting ATPase
7.23	110	2.1	127	NR
6.87	103	1.6	136	NR
6.75	179	1.7	8	NR
6.7	74	6.3	169	Thioredoxin glutathione peroxidase
7.86	110	4.7	118	NR
7.46	110	1.9	126	NR

**NR :** Non recognized

**Table 2:** Recognized proteins of the ES products of *F.* spp. utilizing Expasy bioinformatics database (Expression of proteins in *F. gigantica* more than *F. hepatica* with 95% confidence interval/at the 5% significant level)

***pH***	***Molecular weight (kDa)***	***Fold***	***Spot number***	***Name of protein***
9.39	92	2.5	147	NR
6.27	171	2	20	NR
6.31	180	2.8	6	NR
8.52	110	1.7	123	NR
4.6	127	1.8	91	NR
9.45	169	2	31	NR
4.39	128	1.8	90	NR
6.55	179	2.1	7	NR
6.75	169	1.3	30	NR
8.11	111	3.7	115	NR
4.84	128	1.6	87	NR
7.05	178	2.1	10	NR
6.88	109	1.9	129	Calcium transporting ATPase
7.23	110	2.1	127	NR
6.87	103	1.6	136	NR
6.75	179	1.7	8	NR
6.7	74	6.3	169	Thioredoxin glutathione peroxidase
7.86	110	4.7	118	NR
7.46	110	1.9	126	NR

**NR :** Non recognized

**Table 3:** Recognized proteins of the somatic extract of *Fasciola* spp. utilizing Expasy bioinformatics database (Expression of proteins in *F. hepatica* more than *F. gigantica* with a 95% confidence interval/at the 5% significant level)

***pH***	***Molecular weight (kDa)***	***Fold***	***Spot number***	***Name of protein***
9.39	92	2.5	147	NR
6.27	171	2	20	NR
6.31	180	2.8	6	NR
8.52	110	1.7	123	NR
4.6	127	1.8	91	NR
9.45	169	2	31	NR
4.39	128	1.8	90	NR
6.55	179	2.1	7	NR
6.75	169	1.3	30	NR
8.11	111	3.7	115	NR
4.84	128	1.6	87	NR
7.05	178	2.1	10	NR
6.88	109	1.9	129	Calcium transporting ATPase
7.23	110	2.1	127	NR
6.87	103	1.6	136	NR
6.75	179	1.7	8	NR
6.7	74	6.3	169	Thioredoxin glutathione peroxidase
7.86	110	4.7	118	NR
7.46	110	1.9	126	NR

**NR :** Non recognized

**Table 4:** Recognized proteins of the somatic extract of *Fasciola* spp. utilizing Expasy bioinformatics database (Expression of proteins in *F. gigantica* more than *F. hepatica* with 95% confidence interval/at the 5% significant level)

***pH***	***Molecular weight (kDa)***	***Fold***	***Spot number***	***Name of protein***
4.61	147	5.7	62	NR
6.55	68	7.9	172	Thioredoxin glutathione reductase
5.32	155	2.3	51	NR
6.37	145	2	64	Calcium transporting ATPase
9.12	115	4.9	111	NR
6.58	147	1.9	61	Calcium transporting ATPase
6.52	162	2.4	43	NR
6.99	90	2.1	152	NR
6.75	157	2.1	50	Calcium transporting ATPase
4.08	163	2	40	NR
3.85	163	3.4	42	NR
6.61	186	1.4	2	NR

**NR :** Non recognized

## Discussion

Because of protein importance in parasites, several aspects of these biomolecules were assessed in previous studies such as; Immunogenicity, drug resistance, cell signaling, vaccination and proteomics analysis by IEF and SDS-PAGE (2-DE).

Recent studies confirm ours finding that most proteins concentrate between pH from 4.5 to 6.8 in IEF gel (14). One of the important and vital enzymes for survival of parasites is cathepsin. This enzyme plays many roles such as nutrition, tissue attack, and establishment of worm in bile duct and digestion of food molecules. Besides, host antibodies secreted for defense against parasite could cleaved by cathepsin enzymes of parasites, therefore this enzyme has an excellent role in escape of worm from immune system of host (15). Cathepsin B has important role in invasive to gut wall and migration of worm (16). Despite, cathepsin L cysteine proteinase only were eluted from adult parasites which grew in culture media. Immunohistochemical studies implies this enzyme exists as procathepsin which located in secretory vesicles in digestive tract (17). Our findings revealed cathepsin L and cathepsin B were significantly higher in *F. gigantica* than *F. hepatica*, proteomics study demonstrate cathepsin L in *F. gigantica* was more than *F. hepatica* (18).

In *F. hepatica* leucin aminopeptidase and inulase are two protein which recognized with high immunogenic rate (19). In this research both proteins were detected on 2DE electrophoresis. H_2_O_2_ is oxidized by cytochrome c oxidase. In this reaction, enzyme acts as electron donor. Cytochrome c oxidase is necessary for protection of parasite deoxyribonucleic acid from immune system of host (20). Our results showed cytochrome c oxidase enzyme expression in *F. hepatica* was more than *F. gigantica*.

Triose phosphate isomerase and glyceralde-hyde 3 phosphate dehydrogenase are important metabolic enzymes. Triose phosphate isomerase convert dihydroxyaceton phosphate and glyceraldehyde 3- phosphate each other (21). Our work demonstrate, triose phosphate isomerase enzymes were expressed in *F. gigantica* more than *F. hepatica* and probably the activity of triose phosphate isomerase enzyme in krebs cycle (citrate synthase) was more *in F. hepatica* than *F. gigantica.*

## Conclusion

Some functional and metabolic proteins were detected in two species. The rate of expression of some proteins was more in *F. hepatica* while expression of other proteins was high in *F. gigantica*. The expression of protease enzyme was higher in *F. gigantica* than *F. hepatica*. These data could be considered for biochemical differentiation of *Fasciola* species and subsequently to design and prepare of antigens for diagnosis/vaccine development.
